# Supplementary material for: ThermoMaze behavioral paradigm for assessing immobility-related brain events in rodents
Source: eLife. 2025 Mar 7;12:RP90347. doi: 10.7554/eLife.90347 (PMC11888600; doi:10.7554/eLife.90347)
Supplement: Supplementary file 1. — (a) Summary of animal subjects with brain implants. (b) p-values of multiple group comparisons pertaining to analyses of variance in Figure 3C. Cumulative distribution of animal speed in the ThermoMaze during three sub-sessions. (c) p-values of multiple group comparisons pertaining to analyses of variance in Figure 4. Box plots of Pearson correlation coefficients between spatial firing rate maps. Here, group numbers 1, 2, 3, and 4 refer to correlation values between Pre and Cooling, Cooing and Post, and Pre and Post in control sessions. (d) p-values of multiple group comparisons pertaining to analyses of variance in Figure 5—figure supplement 1A. Pyramidal neurons increase firing rate during ripples in their preferred quadrant during movement. Numbers 1–8 represent pyramidal firing rate: 1. during SPW-R inside the cell’s preferred quadrant during ripple, 2. during SPW-R outside the cell’s preferred quadrant during ripple, 3. during SPW-R inside the cell’s preferred quadrant during movement, 4. during SPW-R outside the cell’s preferred quadrant during movement, 5. during SPW-R inside the cell’s preferred quadrant during ripple, 6. during SPW-R outside the cell’s preferred quadrant during ripple, 7. during SPW-R inside the cell’s preferred quadrant during movement, and 8. during SPW-R outside the cell’s preferred quadrant during movement. (e) p-values of multiple group comparisons pertaining to analyses of variance in Figure 5—figure supplement 1B. Interneurons firing rate does not change during ripples in their preferred quadrant during movement. Numbers 1–8 represent interneuron firing rate: 1. during ripples inside the cell’s preferred quadrant during ripple, 2. during ripples outside the cell’s preferred quadrant during ripple, 3. during ripples inside the cell’s preferred quadrant during movement, 4. during non-ripples outside the cell’s preferred quadrant during movement, 5. during non-ripples inside the cell’s preferred quadrant during ripple, 6. during non-rip [file elife-90347-supp1.docx]

Supplementary Material for

**ThermoMaze behavioral paradigm for assessing immobility-related brain events in rodents**

Mihály Vöröslakos*^1^, Yunchang Zhang*^1^, Kathryn McClain^1^, Roman Huszár^1^, Aryeh Rothstein^1^, György Buzsáki^♰1,2^

*These authors contributed equally to this work.

^1^Neuroscience Institute and ^2^Department of Neurology, School of Medicine, New York University, New York, NY 10016, USA

♰Correspondence: [Gyorgy.Buzsaki@nyulangone.org](mailto:Gyorgy.Buzsaki@nyulangone.org)

**Supplementary File 1a. Summary of animal subjects with brain implants**

| **Animal** | **Recording implant** | **Other implants** | **Behavioral protocol** | **# session** | **Sex** |
| --- | --- | --- | --- | --- | --- |
| Mouse_01 | Diagnostic Biochips, 64-2 | NA | ThermoMaze, 4 corners | 1 | F |
| Mouse_02 | Diagnostic Biochips, 64-2 | NA | ThermoMaze, 4 corners | 3 | F |
| Mouse_03 | NeuroNexus, A5x12-16-Buz-Lin-5mm-100-200-160-177 | NA | ThermoMaze, 4 corners | 1 | F |
| Mouse_04 | Tungsten wire | Thermistor | ThermoMaze, 4 corners | 5 | F |
| Mouse_05 | NA | Thermistor | ThermoMaze, 4 corners | 4 | M |
| Mouse_06 | Diagnostic Biochips, 64-2 | NA | ThermoMaze, 4 corners  Inner spots | 3  2 | F |
| Mouse_07 | NeuroNexus A1x32-Poly3-10mm-25s-177 | NA | ThermoMaze, 4 corners  Inner spots | 3  1 | F |
| Mouse_08 | Cambridge Neurotech 64-ch, F6 | NA | ThermoMaze, 4 corners | 2 | F |
| Mouse_09 | NeuroNexus A1x32-Poly3-10mm-25s-177 | NA | ThermoMaze, 4 corners | 4 | M |
| Mouse_10 | NeuroNexus A1x32-Poly3-10mm-25s-177 | NA | ThermoMaze, sleep | 2 | M |
| Mouse_11 | NeuroNexus A1x32-Poly3-10mm-25s-177 | NA | ThermoMaze, sleep | 1 | F |
| Mouse_12 | NeuroNexus A1x32-Poly3-10mm-25s-177 | NA | ThermoMaze, sleep | 2 | M |
| Mouse_13 | Neuropixels 2.0 | NA | ThermoMaze, sleep | 2 | F |

**Supplementary File 1b.**

| Group A | Group B | p-value |
| --- | --- | --- |
| Pre speed | Cool speed | <0.001 |
| Pre speed | Post speed | <0.001 |
| Cool speed | Post speed | <0.001 |

**Supplementary File 1c.**

| Group A | Group B | p-value |
| --- | --- | --- |
| 1 | 2 | 0.006 |
| 1 | 3 | <0.001 |
| 1 | 4 | <0.001 |
| 2 | 3 | <0.001 |
| 2 | 4 | <0.001 |
| 3 | 4 | <0.001 |

**Supplementary File 1d**.

| Group A | Group B | p-value |
| --- | --- | --- |
| 1 | 2 | <0.001 |
| 1 | 3 | <0.001 |
| 1 | 4 | <0.001 |
| 1 | 5 | <0.001 |
| 1 | 6 | <0.001 |
| 1 | 7 | <0.001 |
| 1 | 8 | <0.001 |
| 2 | 3 | <0.001 |
| 2 | 4 | 0.596 |
| 2 | 5 | <0.001 |
| 2 | 6 | <0.001 |
| 2 | 7 | <0.001 |
| 2 | 8 | <0.001 |
| 3 | 4 | 0.003 |
| 3 | 5 | <0.001 |
| 3 | 6 | <0.001 |
| 3 | 7 | <0.001 |
| 3 | 8 | <0.001 |
| 4 | 5 | <0.001 |
| 4 | 6 | <0.001 |
| 4 | 7 | <0.001 |
| 4 | 8 | <0.001 |
| 5 | 6 | 0.969 |
| 5 | 7 | 0.9999966894 |
| 5 | 8 | 0.9822369191 |
| 6 | 7 | 0.99416517 |
| 6 | 8 | 0.9999999967 |
| 7 | 8 | 0.9973847873 |

**Supplementary File 1e**.

| Group A | Group B | p-value |
| --- | --- | --- |
| 1 | 2 | 0.213 |
| 1 | 3 | 0.76q |
| 1 | 4 | 0.454 |
| 1 | 5 | 0.658 |
| 1 | 6 | 0.663 |
| 1 | 7 | 0.604 |
| 1 | 8 | 0.694 |
| 2 | 3 | 0.988 |
| 2 | 4 | 1.000 |
| 2 | 5 | 0.997 |
| 2 | 6 | 0.996 |
| 2 | 7 | 0.998 |
| 2 | 8 | 0.995 |
| 3 | 4 | 1.000 |
| 3 | 5 | 1.000 |
| 3 | 6 | 1.000 |
| 3 | 7 | 1.000 |
| 3 | 8 | 1.000 |
| 4 | 5 | 1.000 |
| 4 | 6 | 1.000 |
| 4 | 7 | 1.000 |
| 4 | 8 | 1.000 |
| 5 | 6 | 1.000 |
| 5 | 7 | 1.000 |
| 5 | 8 | 1.000 |
| 6 | 7 | 1.000 |
| 6 | 8 | 1.000 |
| 7 | 8 | 1.000 |
